# 3D Object Detection and Instance Segmentation from 3D Range and 2D Color Images †

**DOI:** 10.3390/s21041213

**Published:** 2021-02-09

**Authors:** Xiaoke Shen, Ioannis Stamos

**Affiliations:** 1 The Graduate Center, Computer Science Department, City University of New York, New York, NY 10016, USA; 2 Hunter College & The Graduate Center, Computer Science Department, City University of New York, New York, NY 10065, USA; istamos@hunter.cuny.edu

**Keywords:** frustum, VoxNet, instance segmentation, object detection, 3D CNN, robotics

## Abstract

Instance segmentation and object detection are significant problems in the fields of computer vision and robotics. We address those problems by proposing a novel object segmentation and detection system. First, we detect 2D objects based on RGB, depth only, or RGB-D images. A 3D convolutional-based system, named Frustum VoxNet, is proposed. This system generates frustums from 2D detection results, proposes 3D candidate voxelized images for each frustum, and uses a 3D convolutional neural network (CNN) based on these candidates voxelized images to perform the 3D instance segmentation and object detection. Results on the SUN RGB-D dataset show that our RGB-D-based system’s 3D inference is much faster than state-of-the-art methods, without a significant loss of accuracy. At the same time, we can provide segmentation and detection results using depth only images, with accuracy comparable to RGB-D-based systems. This is important since our methods can also work well in low lighting conditions, or with sensors that do not acquire RGB images. Finally, the use of segmentation as part of our pipeline increases detection accuracy, while providing at the same time 3D instance segmentation.

## 1. Introduction

We are living in a three-dimensional world. Compared to 2D images, 3D images give us a better representation of this world. A better representation of our living world can help automated systems to understand the world with higher certainty. Meanwhile, directly processing the 3D representation of the world can be computationally expensive. Humans can understand the 3D environment in a very efficient way by only focusing on the important parts. For example, it is easy for human beings to realize not salient parts, such as the empty space and background objects, and thus focus on the important objects, such as other people, cars, pets, etc. We want to find a way to use the three-dimensional data more efficiently by simulating human beings’ intelligent behaviors to address the 3D instance segmentation and 3D object detection problem. We achieved this by building a system based on both the 2D and 3D data representations.

Segmentation and object detection are significant problems in the fields of computer vision and robotics. 2D object detection systems from RGB images have been significantly improved in recent years due to the emergence of deep neural networks [1,2,3,4,5], and large labeled image datasets [6,7]. For applications related to robotics though, such as autonomous navigation, grasping, etc., a 2D object detection system is not adequate. Thus, 3D object detection and reconstruction [8] systems have been developed, with input coming from RGB-D or depth-only sensors. We describe a new 3D object detection system that incorporates mature 2D object detection methods as a first step. The 2D detector can run on an input RGB image, or pseudo-RGB image generated from a 3D point cloud. That 2D detection generates a 3D frustum (defined by the sensor and the 2D detected bounding box) where a search for a 3D object is performed. Our main contribution is the 3D object detection and instance segmentation within such a frustum. Our method involves 3D voxelization, not of the whole frustum, but of a learned part of it. That allows for a higher resolution voxelization, lower memory requirements, and a more efficient segmentation and detection.

### 1.1. Problem Definition

#### 1.1.1. 3D Object Detection

Given RGB-D data or depth only data as input, 3D object detection aims to classify and localize objects in 3D space. The depth data, obtained from LiDAR or indoor depth sensors, are represented as a point cloud. Each object is represented by a class (one among *k* predefined classes) and an amodal 3D bounding box. The amodal box is represented by the center (*x*, *y*, *z*), physical size (*w*, *d*, *h*), and orientation (θ,ϕ,ψ) relative to a predefined canonical pose for each category. We only consider the heading angle θ around the up-axis for orientation [9].

#### 1.1.2. 3D Instance Segmentation

The 3D semantic segmentation goal for point cloud is to obtain fine inference by predicting labels for each cloud point. Every cloud point is represented by a class (one among *k* predefined classes). 3D instance segmentation provides different labels for separate instances of objects belonging to the same object-class. Thus, instance segmentation can be defined as the task of simultaneously solving object detection and semantic segmentation [10].

### 1.2. Our Solutions

**Figure 1:**
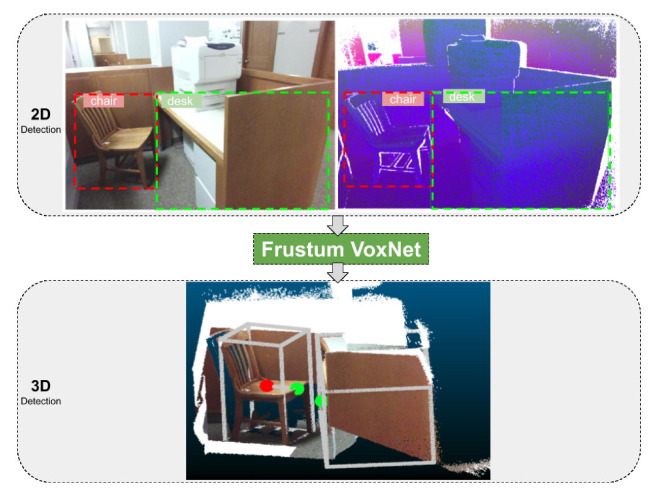
Overview of the whole system. Upper left: RGB image and detected 2D bounding boxes. Upper right: depth height and signed angle (DHS) image, and detected 2D bounding boxes. A DHS image is a pseudo-RGB image generated by a depth image (see text). Bottom: The final 3D detected objects from the associated 3D range image. The 3D detection not only provides an amodal bounding box but also an orientation. The red point is the center of the bounding box and the green one is the front center. The detected 2D bounding boxes from either and RGB or DHS image, generate 3D frustums (which are prisms having as apex the sensor location and extend through the 2D bounding boxes to the 3D space). They are then fed to our Frustum VoxNet network, which produces the 3D detections.

Figure 1 illustrates the overview of our 3D object detection system. In the upper left we see a 2D RGB image, along with the 2D detected bounded boxes (a chair and a desk). On the upper right we see a 2D pseudo-RGB image that was generated from the associated 3D range image (see [11]), along with similarly detected 2D bounded boxes. We call this pseudo-RGB image a DHS image, where D stands for depth, H for height, and S for signed angle. The depth is a normalized distance of the associated 3D point, height is a normalized height of the 3D point, and the signed angle measures the elevation of the vector formed by two consecutive points on a scanline indicating the convexity or concavity of three consecutive points (see [11]). We can apply traditional 2D detectors on this pseudo-RGB image, making our method applicable even when no RGB information is available. 3D frustums are then extracted from these 2D detections. A 3D frustum is a prism having as apex the sensor location and extending through the 2D bounding boxes into the 3D space. Learned parts of the 3D frustum are being voxelized. These voxelizations are fed to Frustum VoxNet, which is a 3D Fully Convolutional Neural Network (FCN), to finalize the 3D object detection.

Our 3D detection is enhanced by our 3D instance segmentation. The voxelizations are fed to a segmentation subnetwork of the Frustum VoxNet, which is a 3D FCN, to produce the 3D instance segmentation.

In this paper, we demonstrate the power of using a 3D FCN approach based on volumetric data to achieve accurate 3D instance segmentation and detection results efficiently. We are presenting a novel method for learning the parts of 3D space to voxelize. This allow us to provide high resolution representations around the objects of interest. It also allows our system to have reduced memory requirements and lead to its efficiency. In addition, compared to systems that do not perform voxelization (such as [9,12]), our methods can operate without the requirement of subsampling the datasets. Compared to systems that do voxelize (such as [13]), our system does not voxelize the whole space, and thus allows a higher resolution object representation. Finally, we provide a 3D instance segmentation and 3D object detection system based on depth only images as well as RGBD images.

In summary our main contributions are as follows:

- We have developed novel methods for 3D objection, classification, and instance segmentation. We have thoroughly tested their efficiency and accuracy as described in Section 3 and Section 4.
- We have significantly improved efficiency with respect to the state-of-the-art in 3D detection. Our 3D detection without segmentation has been presented in [14]. In this paper, we provide an enhanced system that performs both detection and segmentation. That improves the detection performance, and it also includes instance segmentation results. The increased space and time efficiency makes our method appropriate for real-time robotic applications.
- We are able to provide accurate detection and segmentation results using Depth only images, unlike competing methods such as [9]. This is significant, since our methods can also work well in low lighting conditions, or with sensors that do not acquire RGB images.

This paper is organized as follows. Section 2 describes the related work. The object detection system and the experiments based on an indoor dataset are described in Section 3. In Section 4 our 3D instance segmentation system is explained. The 3D object detection based on the 3D instance segmentation output is also described in Section 4. The last section presents our conclusions.

## 2. Related Work

2D methods: RGB-based approaches can be summarized as two-stage frameworks (proposal and detection stages) and one-stage frameworks (proposal and detection in parallel). Generally speaking, two-stage methods such as R-CNN [1], Fast RCNN [2], Faster RCNN [3], Feature Pyramid Network (FPN [4], and mask R-CNN [5] can achieve a better detection performance while one-stage systems such as you only look once (YOLO) [15], YOLO9000 [16], RetinaNet [17], and Single Shot Object Detection with Feature Enhancement and Fusion (FFESSD) [18] are faster at the cost of reduced accuracy. For deep learning-based systems, as the size of the network is increased, larger datasets are required. Labeled datasets such as PASCAL VOC dataset [19] and Common Objects in Context (COCO) [20] have played important roles in the continuous improvement of 2D detection systems. Nice reviews of 2D detection systems can be found in [21,22].

3D methods: Compared with detection based on 2D images, the detection based on 3D data is more challenging due to several reasons [22]: (1) Data representation itself is more complicated. 3D images can be represented by point clouds, meshes, or volumes, while 2D images have pixel grid representations. (2) Due to the extra dimension, there are increased computation and memory resource requirements. (3) 3D data is generally sparser and of lower resolution compared with the dense 2D images, making 3D objects more difficult to identify. Finally, (4) large sized labeled datasets, which are extremely important for supervised-based algorithms, are still inferior compared with well-built 2D datasets. A nice review about different 2D and 3D object detection systems can be found in [22]. Below we summarize the basic approaches.

Project 3D data to 2D and then employ 2D methods: There are different ways to project 3D data to 2D features. Horizontal disparity, height above ground, and the angle the pixel’s local surface normal (HHA) was proposed in [23] where the depth image is encoded with three channels: horizontal disparity, height above ground, and the angle of each pixel’s local surface normal with gravity direction. The signed angle feature described in [24] measures the elevation of the vector formed by two consecutive points and indicates the convexity or concavity of three consecutive points. Input features converted from depth images of normalized depth (D), normalized relative height (H), angle with up-axis (A), signed angle (S), and missing mask (M) were used in [11]. We are using DHS in this work to project 3D depth image to 2D since as shown in [11] adding more channels did not affect classification accuracy significantly. Keeping the number of total channels to three, allow us to use networks with pre-trained weights for starting our training.

2D-driven 3D object detection from RGB-D data: Our proposed framework is mainly inspired by 2D-driven 3D object detection approaches as in [9,25]. First, a 2D detector is used to generate 2D detections. The differences of our work with [25] are: (1) the 2D detector in [25] is only based on RGB images and our proposed system explores both RGB-D and depth only data. (2) 3D detection in [25] uses a MLP regressor to regress the object boundaries based on histograms of points along *x*, *y*, and *z* directions. Converting raw point clouds to histograms results in a loss of information. The main differences of our system to Frustum PointNets [9] are the following: (1) In the 2D detection part, [9] is based on RGB inputs, while our system can support both RGB-D and depth-only sensing. (2) In the 3D detection part, our system is using voxelized data, while Frustum PointNets is consuming raw point clouds via PointNet [12]. PointNet uses a fully connected neural network and max pooling, so it cannot support convolution/deconvolution operations well. We believe 3D convolution/deconvolution can play important roles in both 3D semantic segmentation and object detection. (3) PointNet’s computation complexity is increased if more points are available as the framework’s input is N×K where *N* is the number of points and *K* is the number of channels. (4) Random sampling is required in PointNet, but is not needed in our voxelization approach. A recent method [26] that is based on PointNet and Hough Voting, achieves improved detection results without the use of RGB images. Our method is still more efficient in inference time, and thus more appropriate for robotics application. In addition, our approach does not need to subsample the 3D point cloud as required by [26].

3D CNNs: VoxelNet [13] uses 3D LiDAR data to detect 3D objects based on the KITTI outdoor dataset, and utilizes bird’s eye view (BEV) features (such as Multi-View 3D Object Detection (MV3D) [27], Aggregate View Object Detection (AVOD) [28], and Multi-Modality Sensors of USV [29])). The use of BEV is not helpful in indoor applications. In addition, the use of the whole range image for voxelization lowers the resolution (and therefore the scale) of the objects of interest. Early influential 3D detection systems used two-stage approaches. The first stage generates proposals, while the second stage performs 3D detection. DeepSliding Shape [30] detects 3D objects based on the SUN RGB-D dataset and it uses directional Truncated Signed Distance Function (TSDF) to encode 3D shapes. The 3D space is divided into 3D voxels and the value in each voxel is defined to be the shortest distance between the voxel center and the surface from the input depth map. A fully convolutional 3D network extracts 3D proposals at two scales corresponding to large size objects and small size objects. For the final 3D detection, this method fuses the 3D voxel data and RGB image data by using 3D and 2D CNNs. Our approach, on the other hand, first focuses on the frustum to voxelize, and then selects the part to be voxelized based on training. That allows us to achieve higher resolution around the objects of interest.

3D detection based on multi-sensors: With the development of sensor technologies, multiple sensors can be more available at a reasonable cost. Hence, 3D detection systems based on various sensors are developed in the past several years. In [29] 3D object detection is based on multi-modality sensors of unmanned surface vehicles (USV). Feature deep continuous aggregation (FDCA) [31] aggregates features by using multi-sensors for 3D vehicle detection.

## 3. 3D Object Detection

Object detection is a significant problem in the fields of computer vision and robotics. 2D object detection systems from RGB images have been significantly improved in recent years due to the emergence of deep neural networks and large labeled image datasets. For applications related to robotics though, such as autonomous navigation, grasping, etc., a 2D object detection system is not adequate. Thus, 3D object detection systems have been developed, with input coming from RGB-D or depth-only sensors. In this section we describe a new 3D object detection system that incorporates mature 2D object detection methods as a first step. The 2D detector can run on an input RGB image, or pseudo-RGB image generated from a 3D point cloud. That 2D detection generates a 3D frustum (defined by the sensor and the 2D detected bounding box) where a search for a 3D object is performed. Our main contribution is the 3D object detection within such as frustum. Our method involves 3D voxelization, not of the whole frustum, but of a learned part of it. That allows for a higher resolution voxelization, lower memory requirements, and a more efficient detection.

Figure 1 illustrates the overview of our system. In the upper left we see a 2D RGB image, along with the 2D detected bounded boxes (a chair and a desk). On the upper right we see a 2D pseudo-RGB image that was generated from the associated 3D range image (see [11]), along with similarly detected 2D bounded boxes. We call this pseudo-RGB image a DHS image, where D stands for depth, H for height, and S for signed angle (see Section 1.2). We can apply traditional 2D detectors on this pseudo-RGB image, making our method applicable even when no RGB information is available. 3D frustums are then extracted from these 2D detections. A 3D frustum is a prism having as apex the sensor location and extending through the 2D bounding boxes into the 3D space. Learned parts of the 3D frustum are being voxelized. These voxelizations are fed to Frustum VoxNet, which is a 3D fully convolutional neural network (FCN). We name this system as Frustum VoxNet V1 [14]. The content of this section is reorganized from [14]. All figures and tables in this section are initially from [14].

### 3.1. Dataset

Since our final goal is indoor robotic navigation, our Frustum VoxNet system has been evaluated based on an indoor SUN RGB-D dataset [7]. SUN RGB-D dataset splits the data into a training set which contains 5285 images and a testing set which contains 5050 images. For the training set, it further splits into a training only, which contains 2666 images and a validation set, which contains 2619 images. Similar to [25,30], we are training our model based on the training only set and evaluate our system based on the validation set. We call the only training dataset as train2666 in the future description.

### 3.2. Frustum VoxNet V1 System Overview

First, 2D detections on RGB or DHS image generate 2D bounding boxes of objects. The 2D detections generate 3D frustums (defined by the sensor and the 2D detected bounding box) where a search for a 3D object is performed. For each such frustum, we know the class of the object to be detected by the 2D detection. Our system accurately localizes the amodal 3D bounding box and the orientation of the detected 3D object. To achieve this, we perform 3D voxelization, not of the whole frustum, but a learned part of it. That allows for a higher resolution voxelization, lower memory requirements, and a more efficient detection. We explain first how we decide which part of the frustum to use.

### 3.3. Frustum Voxelization

#### 3.3.1. 3D Cropped Box (3DCB) and 3D Intersection over Itself (IoI)

Given a 3D frustum (defined as a 3D prism from the sensor and the 2D detected bounding box into the 3D space), our goal is to voxelize only a part of it. We define that part as axis-aligned 3D bounding boxes enclosed in the frustum. We call that bounding box a **3D cropped box (3DCB** for short). Given a specific object class (for instance a table), an ideal 3DCB will be big enough to contain all the 3D points belonging to the object, but also small enough to achieve high resolution voxelization. In order to quantify the ability of a given 3DCB to tightly contain a given 3D object, we define the metric 3D Intersection over Itself (IoI). Suppose the object of interest lies in a bounding box 3DBBOX. Then the IoI of the 3DBBOX wrt to a given 3DCB is defined as the volume of intersection of the 3D bounding box with the 3DCB over the volume of the 3D bounding box itself. Therefore an IoI of 1.0 means that the 3DCB is perfectly enclosing the object in 3DBBOX, while as this number tends to 0.0 more and more empty space is included in the 3DCB.

The formula for 3D IoI is:

$$IoI^{3D}=\frac{volume^{3DBBOX}\cap volume^{3DCB}}{volume^{3DBBOX}}$$

From the definition, it is trivial to show that:

$$IoI^{3D}=IoI^{XY}*IoI^{Z}$$

where $IoI^{XY}$ is the IoI in the XY plane and $IoI^{Z}$ is the IoI along the *Z* axis.

$$IoI^{XY}=\frac{area^{3DBBOX^{XY}}\cap area^{3DCB^{XY}}}{area^{3DBBOX^{XY}}}$$

$$IoI^{Z}=\frac{length^{3DBBOX^{Z}}\cap length^{3DCB^{Z}}}{length^{3DBBOX^{Z}}}$$

$3DBBOX^{XY}$ and $3DCB^{XY}$ are 2D projections of 3D bounding box and 3DCB onto the XY plane. $3DBBOX^{Z}$ and $3DCB^{Z}$ are 1D projections of 3D bounding box and 3DCB onto the *Z* axis.

**Figure 2:**
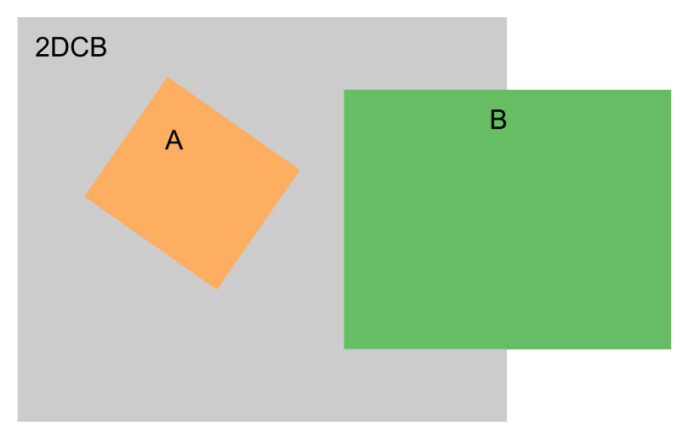
An example of 2D cropped box (2DCB) with two objects box A and box B. All these boxes are square. A has length 1, B has length 2, and 2DCB has length 3. Half of B is overlapped with 2DCB.

We use this metric to choose the optimal 3DCB size. A 2D example in Figure 2 is used to show the difference between IoI and Intersection over Union (IoU). From this example, box A is totally contained in 2DCB (XY plane projection of a 3DCB) while only half of box B is covered by 2DCB. If we use 2D IoU, we will get 0.11 for box A with 2DCB and 0.18 for box B with 2DCB.

#### 3.3.2. Generating 3DCBs Using an IoI Metric and Frustum Voxelization Based on 3DCBs

**Figure 3:**
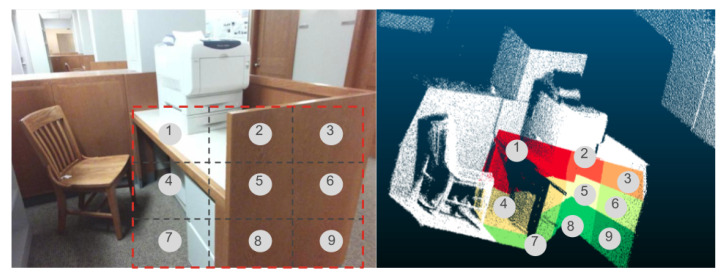
An example of equally subdividing a whole frustum into 3×3 subfrustums (best viewed in color). In this example, the object is a desk. The upper one shows the 2D bounding box of desk is equally divided into 9 small boxes. From each small box, a subfrustum is generated as shown in the bottom image.

During training, given a ground truth 2D bounding box of an object of a given class (for example table) and given the ground truth 3D bounding box of the same object, we would like to calculate the optimal 3DCB. The 3DCB is represented by its center, width, depth, and height. We are adding the constraint that width and depth are the same. This makes sure that the object can freely rotate within the 3DCB along the vertical axis. We proceed by equally dividing the 2D bounding box along the row and column into FR×FC 2D boxes. Then we have FR×FC subfrustums. We will generate FR×FC candidate centers of 3DCBs in that case. The center of each 3DCB is the centroid of the respective frustum. One example of 3×3 subfrustums of a desk is shown in Figure 3. If we set FR=FC=1, then there is only one 3D frustum to consider (and therefore one 3DCB center). Our goal is to calculate the optimal sizes of respective 3DCBs for each object category.

A ground truth 3D bounding box will be recalled (i.e., enclosed into the 3DCB) if the 3D IoI of this box is greater than a threshold. Formally, we define this recall as $recall^{volume}$:

$$recall^{volume}=\frac{|3DCB^{positive}|}{\left|3DCB\right|}$$

where $|3DCB^{positive}|$ is the cardinality of positive 3DCBs and |3DCB| is the cardinality of all 3DCBs. A 3DCB is positive when $IoI^{3D}=IoI^{XY}*IoI^{Z}\geq threshold$. To make the parameter setting simple, we are exploring the recall of XY plane and *Z* axis separately. Similar to $recall^{volume}$, $recall^{XY}$ and $recall^{Z}$ are defined as: $recall^{XY}=\frac{|3DCB_{XY}^{positive}|}{\left|3DCB\right|}$, $recall^{Z}=\frac{|3DCB_{Z}^{positive}|}{\left|3DCB\right|}$, where $|3DCB_{XY}^{positive}|$ is the cardinality of positive 3DCBs in XY plane, $|3DCB_{Z}^{positive}|$ is the cardinality of positive 3DCBs in Z axis and |3DCB| is the cardinality of all 3DCBs. A 3DCB is positive in XY plane when $IoI^{XY}\geq threshold_{XY}$ and a 3DCB is positive in *Z* axis when $IoI^{Z}\geq threshold_{Z}$.

Although, we cannot naively have $recall^{volume}=recall^{XY}*recall^{Z}$, we have a nice inequality to guarantee a lower bound of $recall^{volume}$:

(1) $$recall^{volume}\geq \max\left(0,\;recall^{XY}+recall^{Z}-1\right)$$

The proof of this inequality is given below:

**Proof.**  Define the threshold used for positive 3DCB as $threshold_{3D}$, and a 3DCB is positive when $IoI^{3D}=IoI^{XY}*IoI^{Z}\geq threshold_{3D}$. The $recall^{volume}$$recall^{XY}$, $recall^{Z}$, $threshold_{XY}$ and $threshold_{Z}$ are defined in the main article. We set the $threshold_{3D}=threshold_{XY}*threshold_{Z}$.

As $3DCB_{XY}^{positive}\cap 3DCB_{Z}^{positive}$ implies $IoI^{XY}\geq threshold_{XY}$ and $IoI^{Z}\geq threshold_{Z}$. We can further get $IoI^{3D}=IoI^{XY}*IoI^{Z}\geq threshold_{XY}*threshold_{Z}=threshold_{3D}$, which implies 3DCBs in the set of $3DCB_{XY}^{positive}\cap 3DCB_{Z}^{positive}$ are positive. Meanwhile, we can show from an example that the set of $3DCB^{positive}$ can possibly be obtained from $3DCB_{XY}^{positive}\cap 3DCB_{Z}^{nonpositive}$, where $3DCB_{Z}^{nonpositive}$ is a complement set of $3DCB_{Z}^{positive}$: if $threshold_{XY}=0.9,\;threshold_{Z}=0.9$, we can get $threshold_{3D}=0.81$. A 3DCB with $IoI_{XY}=1.0,\;IoI_{Z}=0.82$ will be an element of set $3DCB_{XY}^{positive}\cap 3DCB_{Z}^{nonpositive}$. Also it is a positive 3DCB. From above arguments, we can conclude the following relation:

(2) $$3DCB^{positive}⊇\left\{3DCB_{XY}^{positive}\cap 3DCB_{Z}^{positive}\right\}$$

From Equation (2), we can get:

(3) $$|3DCB^{positive}|\geq |3DCB_{XY}^{positive}\cap 3DCB_{Z}^{positive}|$$

We can also rewrite right part of Equation (2) as:

(4) $$\begin{array}{cc} \{3DCB_{XY}^{positive} & \cap \;3DCB_{Z}^{positive}\}\\ \; & =3DCB_{XY}^{positive}\setminus \\ \; & \;\{3DCB_{XY}^{positive}\cap \left\{3DCB\setminus 3DCB_{Z}^{positive}\right\}\} \end{array}$$

From Equation (4), we can further get:

(5) $$\begin{array}{cc} \; & |3DCB_{XY}^{positive}\setminus \\ \; & \;\{3DCB_{XY}^{positive}\cap \left\{3DCB\setminus 3DCB_{Z}^{positive}\right\}\}|\\ \; & \;\geq |\{3DCB_{XY}^{positive}\setminus \left\{3DCB\setminus 3DCB_{Z}^{positive}\right\}|\\ \; & \;\geq |3DCB_{XY}^{positive}|-|3DCB\setminus 3DCB_{Z}^{positive}|\\ \; & \;=|3DCB_{XY}^{positive}|-(|3DCB|-|3DCB_{Z}^{positive}\left|\right) \end{array}$$

From Equations (3) and (5), we can get:

(6) $$\begin{array}{cc} |3DCB^{positive}| & \geq |3DCB_{XY}^{positive}|-(|3DCB|-|3DCB_{Z}^{positive}\left|\right)\\ \; & =|3DCB_{XY}^{positive}|+|3DCB_{Z}^{positive}\left|-|3DCB\right| \end{array}$$

From Equation (6), we can get:

(7) $$\begin{array}{cc} \; & \frac{|3DCB^{positive}|}{\left|3DCB\right|}\\ \; & \;\geq \frac{|3DCB_{XY}^{positive}|+|3DCB_{Z}^{positive}\left|-|3DCB\right|}{\left|3DCB\right|} \end{array}$$

Equation (7) can be rewritten as:

(8) $$recall^{volume}\geq recall^{XY}+recall^{Z}-1$$

Since $recall^{volume}$ is supposed to be greater or equal to 0, we get:

(9) $$recall^{volume}\geq \max\left(0,\;recall^{XY}+recall^{Z}-1\right)$$

□

**Figure 4:**
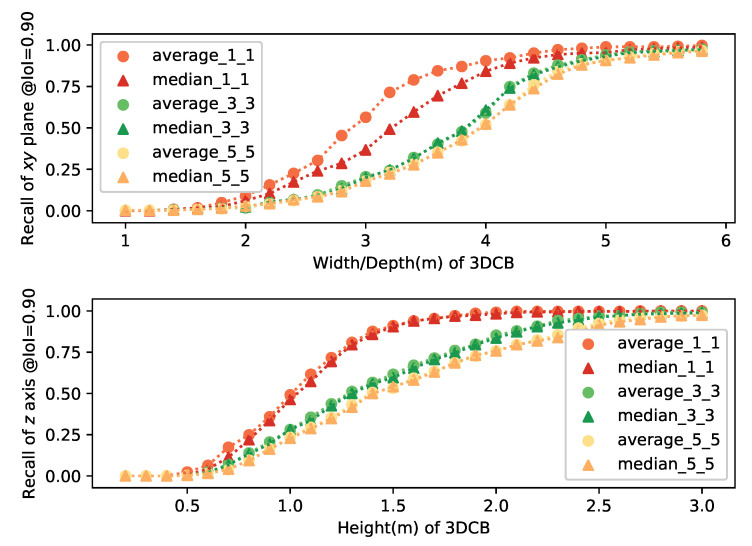
$IoI^{XY}$ and $IoI^{Z}$ with the widths/depths and heights. 3D cropped boxes (3DCBs) are generated from average/median center based on FR×FC subfrustums with different widths/depths and heights. In this plot, verage/median_m_n corresponds to recall based on average/median center in m×n subfrustums.

Both the $threshold_{XY}$ and $threshold_{Z}$ are set as 0.90. We are generating both the average center and median center from subfrustums and pick up the best one from these FR×FC candidates to calculate the recall. The average recall based on different setups of width/depth and height are shown in Figure 4. From the results, we can observe: (1) the performance of the average center based 3DCB is better especially when 1×1 subfrustums are used compared with the median center. The reason for this might be the range of indoor depth sensor is limited and outliers will not have too much influence to the results. (2) The 3DCB generated from 1×1 is better than 3×3 and 5×5 ones. Based on these observations, we are choosing both 1×1 and 3×3 during training to generate more samples and make the training robust to the inaccurate bounding box predictions. During inference, 1×1 subfrustum-based 3DCB is used to speed up and get better performance.

The generated 3DCBs are voxelized to finish the frustum voxelization process.

### 3.4. Double Frustum Method

To increase the accuracy of the center calculations, we developed a double frustum framework. We use a smaller 2D bounding box to generate a smaller frustum for the calculation of the 3DCB center. The estimated center should now be more accurate since it will concentrate on the central part of the object and thus will avoid the use of other background objects. A 3DCB is then selected from a larger frustum in order to contain background context points and possible false negative points. The larger frustum is generated from a larger 2D bounding box. During training, we generate a large frustum by randomly increasing the 2D bounding box width and height by 0% to 15% independently. For the small frustum, we randomly decrease the 2D bounding box width and height by 0% to 10% independently. During inference, the large frustum is generated by increasing the 2D bounding box width and height by 5%. Original 2D detection bounding boxes are used to calculate the 3DCB center.

### 3.5. Multiple Scale Networks

In [30], two scales network were used for different categories concerning the 3D physical size. We are using 4 scales networks to voxelize the 3D objects corresponding to the average physical size of average height, maximum of average width, and depth. The mapping of 3D object categories to different scales is shown in Table 1.

**Figure 5:**
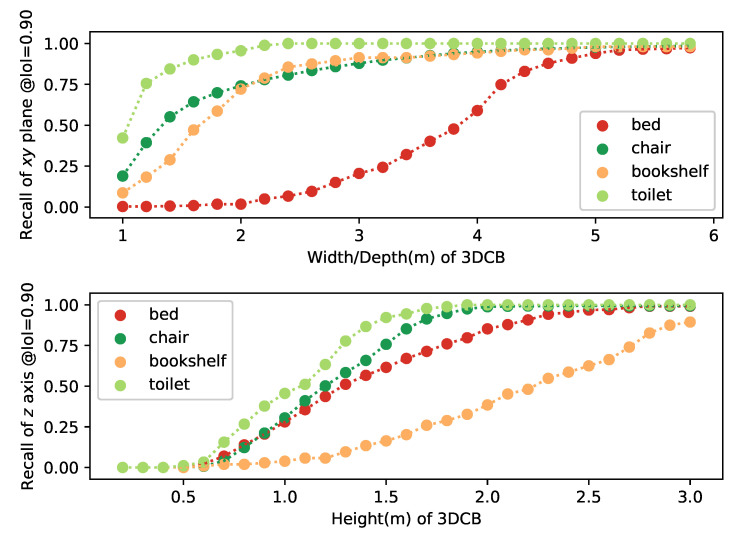
XY plane recall and *Z* axis recall for bed, chair, bookshelf, and toilet with the widths/depths and heights based on train2666 dataset.

We are calculating the $recall_{XY}$ and $recall_{Z}$ for different objects with the different setups for width/depth and heights. The curves of $recall_{XY}$ with width/depth and $recall_{Z}$ with height are plotted for four classes based on 3×3 subfrustums (sofa is from large short scale, chair is from medium short scale toilet is from small short scale and bookshelf is from median tall scale) are shown in Figure 5. From these curves, we can find out that medium tall scale category needs greater height and both the large short and medium short categories need more width/depth. We are selecting the minimum width/depth and height which can guarantee all objects within that scale network can meet the requirements of $recall^{XY}\geq 0.90$ and $recall^{Z}\geq 0.95$. This is based on 3×3 subfrustums. From Equation (1), we can have the lower bound of the $recall_{volume}$ of 0.85. Although 0.85 is not high enough, when based on 1×1 subfrustums, the lower bound of the $recall_{volume}$ can achieve 0.94 as $recall^{XY}\geq 0.95$ and $recall^{Z}\geq 0.99$ for 3DCBs generating from 1×1. Since we are using both 3DCBs from 1×1 and 3×3 subfrustums, the recall is good enough to support the training.

The physical sizes (width/depth/height) of 4 scale networks are shown in Table 2 based on the principles described above. 3DCB are further voxalized (counting the number of cloud points within each voxel) into a 3D tensor with the shape of W×D×H. The W×D×H for each scale network are selected to make it having a better resolution as compared with [30]. The comparison of physical size, resolution, tensor shape of the RPN, and detection networks of [30] and ours are also shown in Table 2.

### 3.6. 3D Object Detection

#### 3.6.1. 3D Bounding Box Encoding

Similar to [30], we are using the orientation, center, width, depth, and height to encode the 3D bounding box.

#### 3.6.2. Detection Network Architecture

**Figure 6:**
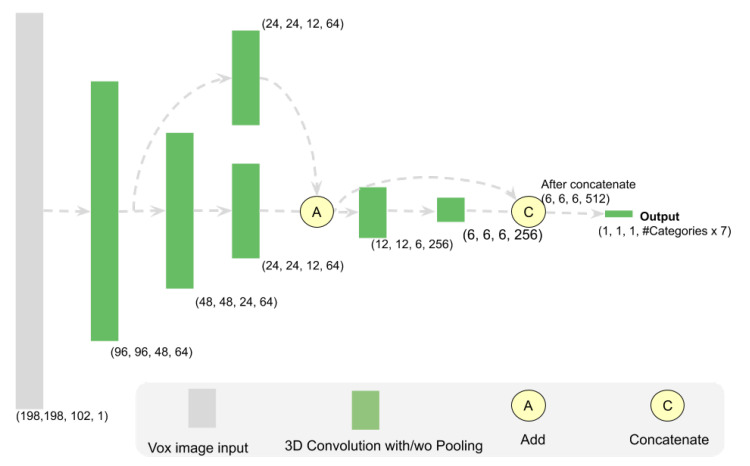
ResnetFCN6 architecture (used for large short scale). Every 3D CNN layer will be followed by a dropout layer. The tensor shape shown here is the output shape of each block. It provides the (width, depth, height, channel) information of the network. The other three scale networks have the same structure with different input size as shown in Table 2.

**Figure 7:**
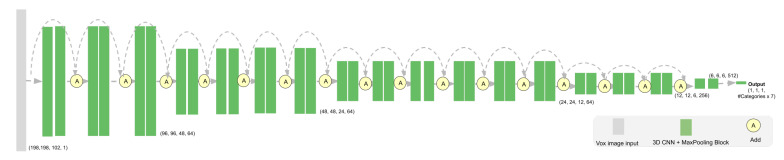
ResNetFCN35 network structure.

We are using 3D FCN networks to build the 3D detection network by adapting the network structure of ResNet [32], and fully convolutional network (FCN) [33]. We propose a fast 6 layer fully convolutional 3D CNN model as shown in Figure 6. We name it ResnetFCN6 with respect to ResNet [32] and FCN [33]. We also tried the ResNetFCN35, which has 35 3D CNN layers. The network structure is shown in Figure 7. Since the ResnetFCN6 can already provide us good results and the inference speed is faster, we use ResnetFCN6 as our main network for 3D detection.

Inputs of our networks are voxelized images. Our network will have C∗7 outputs, where *C* is the number of classes within the corresponding scale network, and 7 is the orientation, center xyz, and size (width/depth/height) predictions. The 2D prediction info is implicitly encoded in the system since the prediction is based on each category.

#### 3.6.3. Loss Function

We are generating loss function for detection by adjusting the loss function from YOLO9000 [16]. Similar to [16], we use simple L2 distance instead of Kullback–Leibler divergence to evaluate the difference of predicted category probability distributions and the ground truth distributions. For the regression part, for centers, we normalize the x,y,z values to 0 and 1 and then use a sigmoid function to make the prediction. For width (*w*), depth (*d*), and height (*h*), we use anchor to support the prediction. For each category, we set the anchor as the average value of the train2666 samples for objects within this category. The ratio of the bounding box to the related anchors are used to drive the network to make the correct prediction. The formal definition of the loss is given in the formulas below.

$$L_{detection}^{3D}=\lambda_{1}L_{orientation}+\lambda_{2}L_{xyz}+\lambda_{3}L_{wdh}$$

where $L_{xyz}=L_{x}+L_{y}+L_{z},\;L_{wdh}=L_{w}+L_{d}+L_{h}$, $L_{x}={\left(x-x^{★}\right)}^{2}$, $L_{y}={\left(y-y^{★}\right)}^{2}$, $L_{z}={\left(z-z^{★}\right)}^{2}$, $L_{w}={\left(\log\frac{w}{a_{w}}-\log\frac{w^{★}}{a_{w}}\right)}^{2}$, $L_{d}={\left(\log\frac{d}{a_{d}}-\log\frac{d^{★}}{a_{d}}\right)}^{2}$, $L_{h}={\left(\log\frac{h}{a_{h}}-\log\frac{h^{★}}{a_{h}}\right)}^{2}$. $a_{w}$, $a_{d}$, $a_{h}$ are width/depth/height of anchors. $\lambda_{1}$, $\lambda_{2}$, $\lambda_{3}$ are used to balance losses.

By combining the loss of orientation, center (xyz), and the physical size (width/depth/ height) together, our system can learn to regress the 3D bounding box of the target object through each part. In addition, the combined loss makes the training process very efficient.

### 3.7. Training Process and Data Augmentation

For the 2D detection, we are using ResNet [32] 101 layer as the backbone and using the feature pyramid layers proposed by [4] which is based on Faster RCNN [3] approach. The loss is the same as [4]. For the 2D detection, the network is pretrained on COCO dataset. Then it is retrained on SUN RGB-D dataset based on RGB or DHS images. Although, the DHS images are different to the RGB images, we find the pretrained weights can still speed up the whole training process and improve the detection results. Data are augmented by adding Gaussian blur, random cropping, and image translating up to 10% of the original images.

For the 3D detection, we use the stochastic gradient descent (SGD) with learning rate of 0.01 and a scheduled decay of 0.00001. For regulation we use batch normalization [34]. The cloud points are randomly rotated around z-axis and jittered during the voxelization process before feeding them to the network.

### 3.8. Efficiency Boost by Pipelining

**Figure 8:**
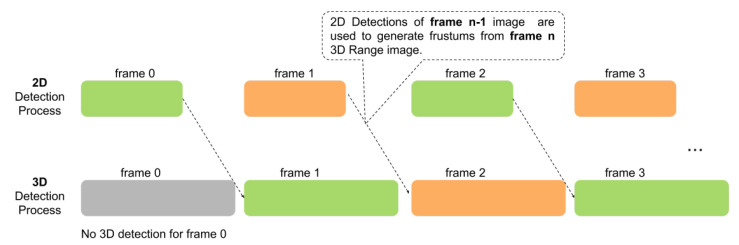
Illustration of using pipelining to speedup the whole detection framework.

Pipelining instructions is a technology used in central processing units to speed up the computing. An instruction pipeline reads an instruction from the memory while previous instructions are being executed in other steps of the pipeline. Thus, multiple instructions can be executed simultaneously. Pipelining can be perfectly used in our system as we have two stages, one is 2D detection and one is 3D detection. In the 3D detection, instead of using the 2D detection of frame n, we can use the 2D detection results of frame n−1 and generate frustums based on that. By using pipelining, our system can be sped up from $t_{2D}+t_{3D}$ to $\max(t_{2D},t_{3D})$, where $t_{2D}$ and $t_{3D}$ are the 2D and 3D detection time, respectively. The disadvantage of using pipelining is frustums generated from the previous 2D image maybe not accurate under the fast movement of the sensor of an object of interest. However, our system will not suffer significantly as our results show, due to robustness on frustum location. We use multiple candidates with different centers during training to make it robust. Meanwhile, the double frustum method used in our system makes our 3D detections robust to slightly moved 2D detections. The illustration of the pipelining method is shown in Figure 8. By using pipelining, our system can be sped up to 48 ms (this is about 2.5× speedup to the state-of-the-art [9]) when use YOLO v3 and ResNetFCN6. It can achieve 21 frames per second which can well support real time 3D object detection.

### 3.9. Experimental Results for the Frustum VoxNet V1 System

#### 3.9.1. Effects of Batch Normalization, Group Normalization, and Dropout

**Figure 9:**
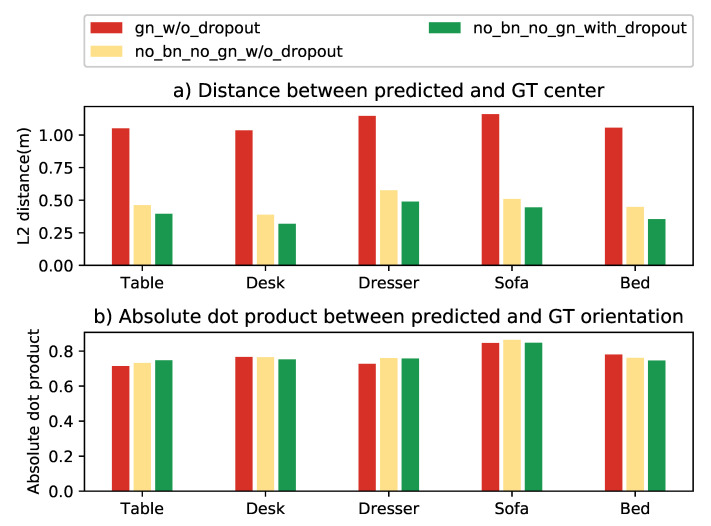
Performance comparison of different combinations on using batch normalization (BN), group normalization (GN) and dropout. “gn_w/o_dropout” means using GN without dropout. “no_bn_no_gn_w/o_dropout” means using none. “no_bn_no_gn_with_dropout” means not using BN/GN, however, the dropout is used.

Overfitting can be an issue for supervised machine learning-based systems as it will have poor performance in the test stage, although the system performs well during the training process. Dropout [35] is a powerful tool to prevent neural networks from overfitting. Batch normalization (BN) [34] is another method we can use to speed up the training and also prevent overfitting. However, BN performs better when the batch size is large enough. Since Frustum VoxNet is using 3D CNNs based on Voxelized images, large batch sizes are not well supported when single GPU is used. Some new technologies are introduced to address the small batch size problem such as group normalization (GN) [36]. We explore the performance of different combinations of these methods by evaluating the performance of center and orientation predictions. Results are shown in Figure 9. We do not use BN as our batch size is small and the using of BN will lead to inconsistencies between training and inference. Although when using the GN, there are no inconsistencies between training and inference, the performance of center prediction is worse compared with not using any normalization. Therefore, our final model does not use any normalization. However, dropout is used in our final model as it can improve the center prediction performance.

The dropout’s better performance shows the power of this simple but efficient method for addressing the overfitting issue. There are many explanations of why dropout works. I did not find anyone mentions that the dropout process has some similarities to ensemble methods. In machine learning, ensemble methods use multiple learning algorithms to obtain better predictive performance than could be obtained from any of the constituent learning algorithms alone [37]. In the training process, dropout randomly drop units (along with their connections) from the neural network. This generates different ’thinned’ networks. At test time, it is easy to approximate the effect of averaging all these thinned networks’ predictions by using a single unthinned network with smaller weights [35]. At the test time, the averaging process is kind of ensembling the output of different networks to achieve better results and hence overcome the overfitting.

#### 3.9.2. Evaluation of the Whole System

First we evaluate the 2D detector in Table 3. The evaluation is based on the standard mAP metric with IoU threshold of 0.5. Comparing our RGB-based and depth-based (DHS image) 2D detections, we see that in most cases RGB performs better, but the depth-based 2D detector is competitive. For few classes such as bathtubs, DHS results are slightly better. The reason might be that some classes such as bathtubs have special geometric shapes and they are easier to be detected by depth sensors. Comparing with state-of-the-art methods, our 2D detector performs better in some categories, and we are also introducing new categories. We are on par with most other categories, except for bathtub, desk, and bookshelf.

Full 3D detection results are shown in Table 4. We provide various variations in our system. First two variations include RGB 2D detector, and the last two include depth only (DHS) 2D detector. In all cases, we use an FPN for the 2D detector. For the 3D detection we have experimented with ResNetFCN6 and ResNetFCN35. As in the 2D case, our 3D detector is on par in most categories with the state-of-the-art, and we have also incorporated more classes. Looking at the computational performance of the 3D detector only, we see that our implementation using ResNetFCN6 provides significant improvements on inference time. Since the architecture is modular (i.e., we can swap out our 2D detector with one from the reported as state-of-the-art), we see that our approach can lead to significant efficiency improvements, without a significant drop in detection accuracy. That will lead to a system geared to real-time robotics applications.

We have also evaluated the efficiency and accuracy of our system when a very fast 2D detector (YOLO v3) is being used. Table 5 shows the decrease in detection accuracy as expected. Finally Table 6 provides a detailed analysis of multiple network combinations in terms of efficiency, along with the number of parameters to tune. As mentioned before, we can achieve faster inference times in 3D detection, and can thus lead to a faster system overall if we swap our 2D detector with the ones reported as state-of-the-art. Using YOLO and pipelining approaches, we can provide a significant boost in total efficiency, with accuracy loss though.

### 3.10. Evaluate Frustum VoxNet Results Based on Ground Truth 2D Bounding Box

#### 3.10.1. Orientation Results

**Figure 10:**
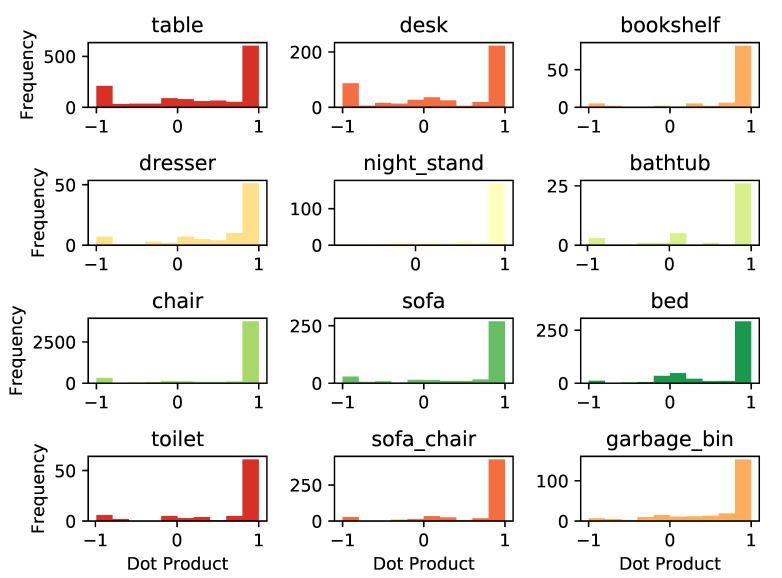
Histogram of the dot product between predicted orientation and ground truth orientation. Histograms are not normalized.

We use the dot product between the ground truth orientation and predicted one to evaluate the orientation prediction performance. If the dot product is 1, the prediction is perfect. If it is −1, it means that we have a flipped prediction. Histograms of the dot product between predicted orientations and ground truth orientations for each category are shown in Figure 10. From the results, we can see for most categories that we have a pretty good orientation prediction. For some categories, such as table and desk, the orientation is flipped.

#### 3.10.2. Bounding Box Center, Physical Size, and 3D Detection Results

We use the following metrics to evaluate the predictions for the center and physical size of the bounding box based on ground truth 2D bounding boxes.

$$D_{x}=|x^{*}-x|,\;D_{y}=|y^{*}-y|,\;D_{z}=\left|z^{*}-z\right|$$

$$D_{w}=|w^{*}-w|,\;D_{d}=|d^{*}-d|,\;D_{h}=\left|h^{*}-h\right|$$

$$D_{xyz}=\sqrt{{\left(x^{*}-x\right)}^{2}+{\left(y^{*}-y\right)}^{*}+\left(z^{*}-z\right)}|$$

$$D_{wdh}=\sqrt{{\left(w^{*}-w\right)}^{2}+{\left(d^{*}-d\right)}^{*}+\left(h^{*}-h\right)}|$$

$x^{*},y^{*},z^{*}$ are the predicted center and x,y,z are ground truth. $w^{*}$, $d^{*}$, $h^{*}$ are the predicted width/depth/height and w,d,h are ground truth.

We compare the center prediction based on the frustum average center and the prediction from our Frustum VoxNet system. Table 7 provides the average distance between predicted and ground truth centers by using these two methods. As expected, the Frustum VoxNet prediction is better than the average center from frustum.

**Figure 11:**
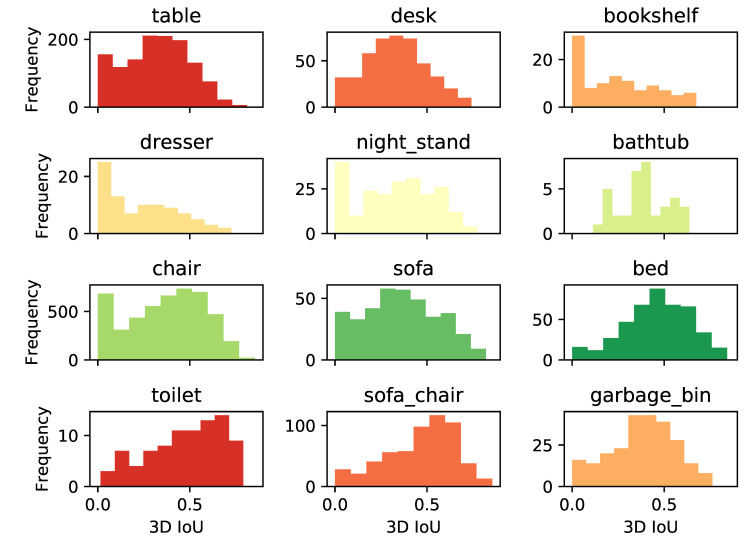
Histogram of 3D IoU. Histograms are not normalized.

Evaluation results for the performance of Frustum VoxNet based on frustums generated from ground truth bounding boxes are shown in Table 8. Histograms of 3D detection IoU for each category are shown in Figure 11.

### 3.11. Visualizations of 2D and 3D Detection Results

**Figure 12:**
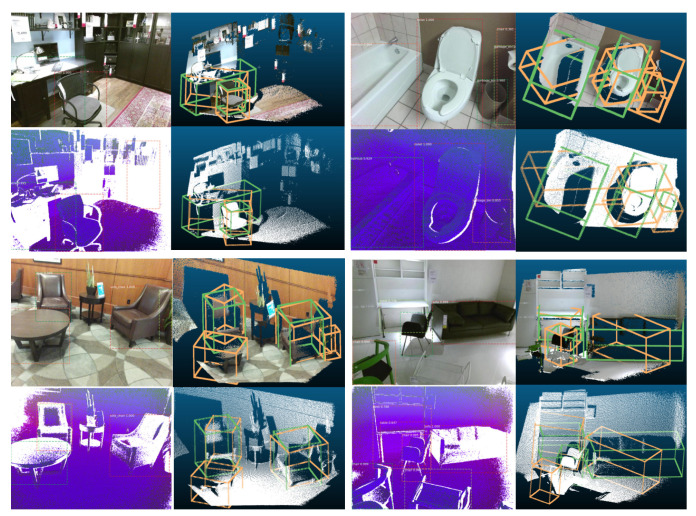
Visualizations of 2D and 3D detection results part 1. This visualization contains four RGB images and four DHS images. For each image, 2D detection is shown superimposed. Next to each image we show the corresponding 3D detection results (light green ones are the 3D ground truth boxes and orange-colored boxes are the predictions) based on frustums generated from image 2D detections (to have a better visualization, RGB colors are projected back to the cloud points).

**Figure 13:**
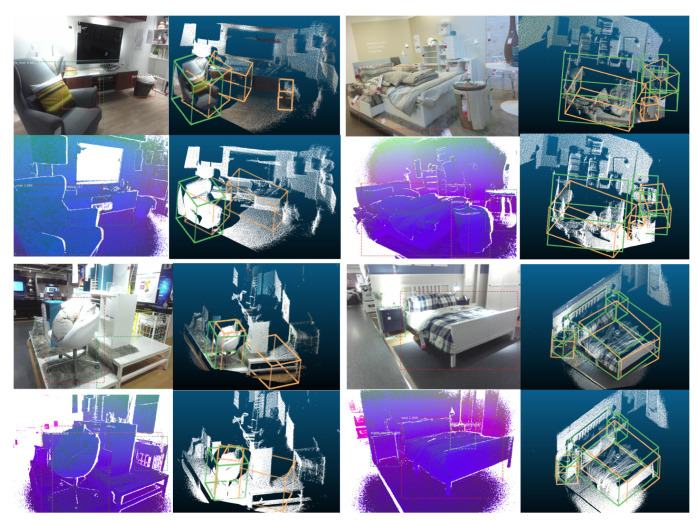
Visualizations of 2D and 3D detection results part 2. Please read the caption of Figure 12 to get an explanation about how to understand the visualization.

Visualizations of both 2D and 3D detection results are shown in Figure 12 and Figure 13 for both the based on RGB-D system and based on depth image only system. For those two figures, the upper right shows the corresponding 3D detection results (light green ones are the 3D ground truth boxes and orange-colored boxes are predictions) based on frustums generated from RGB image 2D detections (to have a better visualization, RGB colors are projected back to the cloud points). Lower left shows 2D detection based on DHS image. Lower right shows the corresponding 3D detection results (light green ones are the 3D ground truth boxes and orange-colored boxes are predictions) based on frustums generated from DHS image 2D detections. From Figure 12, for the first image in the first row, our system can perfectly detect the chair. For the desk, the orientation is off as the frustum generated by the 2D bounding box contains some cloud points from the chair. For the second image, we can see that the based on RGB image system detect more false positive objects in the 2D stage and hence more 3D false positive objects will be detected. For the first image of the second row, our system successfully detect the unlabeled table. For the last image, the sofa’s orientation is off as there are too many points are missing for the sofa. From Figure 12 we can see that the 3D detection system works well for both the based on RGB-D and based on depth only systems. The RGB-D-based 3D detection system will generate some false positive 3D detections as it has more false positive detection during 2D detection stage. We can also find out that our system can detect objects which were not labeled during the data annotation. In Figure 13, on the left part, our system can successfully detect unlabeled objects such as garbage bin and table. On the top right image, our system fails to detect one table in 2D detection stage as it is partially observed. For the last one, one night stand is undetected as it is blocked by bed.

## 4. 3D Instance Segmentation and Object Detection

**Figure 14:**
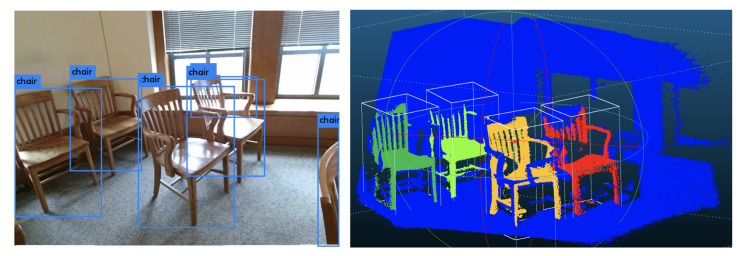
An example of 3D instance segmentation. The left image is the RGB color image and the right is the instance segmentation based on the point cloud. The image comes from the SUN RGB-D dataset and the instance segmentation visualization is based on the ground truth 3D segmentations.

Different from the 2D instance segmentation, 3D instance segmentation is more natural. As the 2D image consists of projections of 3D objects, different items may overlap with each other in the 2D image plane. However, in the 3D space, different objects are separated naturally. Figure 14 shows a visualization of the 3D instance segmentation from the SUN RGB-D dataset. From the visualization, we can see that different chairs are separated naturally in the 3D space. However, in the 2D image plane, we can not efficiently separate chairs from each other. Based on this observation, we propose our Frustum VoxNet V2, which introduces the 3D instance segmentation in our system. 3D detection based on 3D instance segmentation can significantly boost the final performance.

### 4.1. Overview of the Frustum VoxNet V2 System

**Figure 15:**
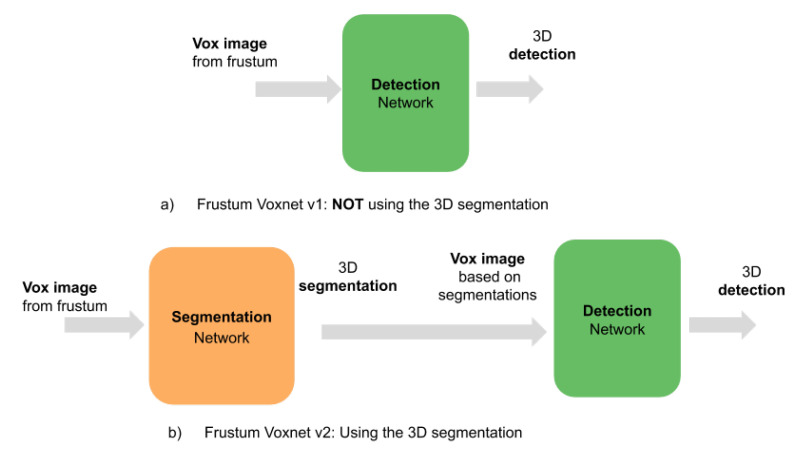
Frustum VoxNet V1 vs. Frustum VoxNet V2.

The Frustum VoxNet V2 system is also a 2D driven system. The differences with the Frustum VoxNet V1 are as follows. V2 supports 3D instance segmentation, and its 3D detection is based on the output of the 3D instance segmentation. The 2D detection part for Frustum VoxNet V2 is identical to V1. For the 3D part, Figure 15 shows the difference between V1 and V2. For the V2 system, we have two steps: (1) We feed the voxelized image (generated by the frustum voxelization process) to a segmentation network to generate 3D instance segmentation. (2) The frustum will be voxelized based on the 3D instance segmentation, and then this voxelized image is fed to the 3D detection network to produce the 3D object detection.

### 4.2. 3D Instance Segmentation

#### 4.2.1. Instance Segmentation Network Architecture

**Figure 16:**
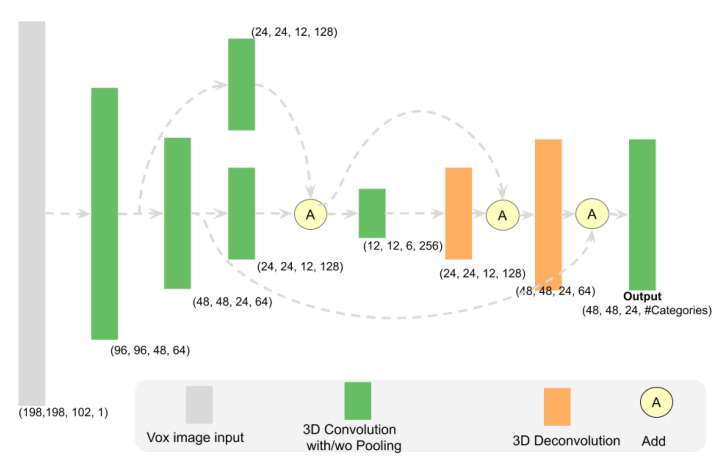
Segmentation architecture (used for large short scale). Every 3D CNN layer will be followed by a dropout layer. The tensor shape shown here is the output shape of each block. It provides the (width, depth, height, channel) information of the network. The other three scale networks have the same structure with different input size as shown in Table 2.

The segmentation network architecture (see Figure 16) is adjusted from the ResNetFCN6 in Frustum VoxNetNet V1. It has the same input as the ResNetFCN6. However, the output is a tensor with the shape of (width, depth, height, #categories) as we are predicting the instance segmentation mask per category. Table 9 shows the detailed input and output shapes for the segmentation network.

#### 4.2.2. Segmentation Ground Truth Based on Voxelization

The original ground truth from the SUN RGB-D dataset is per cloud point. Since our system’s output is a 3D voxelized image (a 3D tensor), we convert the original per cloud point mask into a 3D binary voxelized image. We first generate a volumetric representation encodes the 3D cloud point as a 3D tensor of real values (the real value here means how many cloud points are dropping inside of a voxel). Then we change the 3D tensor of real values to 3D tensor of binary values by using the thresholding method. Specifically, we create the 3D tensor of binary value by using the following formula:

$$f\left(x\right)=\left\{\begin{array}{cc} 1, & \mathrm{if}\;\mathrm{voxel}\;\mathrm{is}\;\mathrm{not}\;\mathrm{empty}\;\mathrm{and}\;\mathrm{positive}\;\mathrm{point}\;\mathrm{ratio}\geq \;Threshold\\ 0, & \mathrm{otherwise} \end{array}\right.$$

#### 4.2.3. Segmentation Loss Function

Similar to the 2D instance segmentation loss in the Mask R-CNN [39], we are using a sigmoid function to predict the 3D mask and the loss is using the binary cross-entropy loss. As more than 97% of the voxels are empty, we only apply loss to non empty voxels. Specifically, we are using the following formula.

$$CE_{(}gt^{v_{\left(i,j,k\right)}},p^{v_{\left(i,j,k\right)}})=-\sum_{x\in \{0,1\}}gt_{x}^{v_{\left(i,j,k\right)}}*p_{x}^{v_{\left(i,j,k\right)}}$$

$$Loss=\sum_{i=0}^{W}\sum_{j=0}^{D}\sum_{k=0}^{H}CE_{(}gt^{v_{\left(i,j,k\right)}},p^{v_{\left(i,j,k\right)}}){⊮}_{A}\left(v_{\left(i,j,k\right)}\right)$$

where W,D,H are the size of the segmentation output tensor. $v_{(i,j,k)i}$ is a voxel with index of i,j,k. $gt^{v_{\left(i,j,k\right)}}$ is the ground truth of the mask for this voxel and $p^{v_{\left(i,j,k\right)}}$ is the prediction probability of the voxel being positive or negative. *A* is a set contains all non empty voxels of this image.

### 4.3. 3D Object Detection

#### 4.3.1. 3D Object Detection Network Architecture and Loss Function

3D object detection network architecture and loss function of V2 are identical to V1.

#### 4.3.2. 3D Object Detection Network Inputs

For V2, the training uses the voxelized image based on the ground truth of the 3D segmentation. For the inference, the input is the voxelized image based on the predicted 3D segmentation.

### 4.4. Training Process

**Figure 17:**
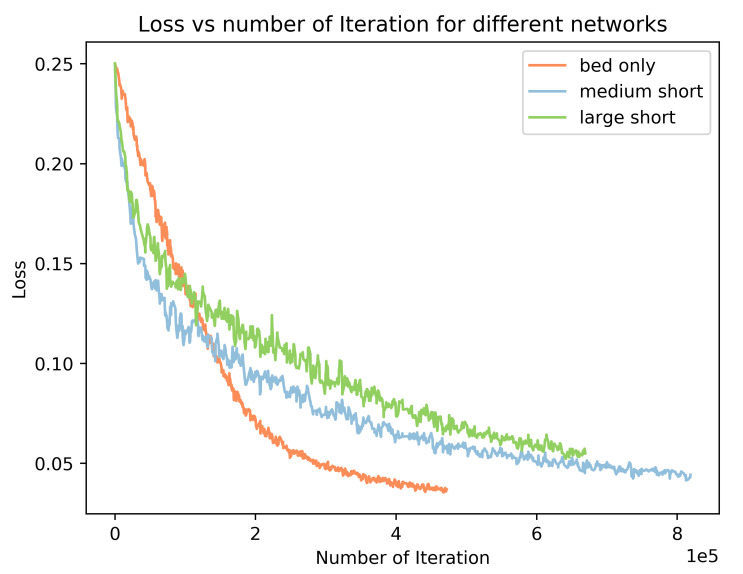
Segmentation training loss for different scale networks.

We use the Stochastic Gradient Descent optimizer to train both the segmentation and detection network. The training loss for each network can be found in Figure 17. From the result, we can see the training process converge smoothly with the increasing of the number of the iterations. In addition, when the training task has less samples, the training takes less iterations. For example, the number of instances for the bed only is a subset of the large short network (bed, sofa, table, desk, and dresser), and the training of the bed only network can converge faster than the large short network: the training of the large short network needs 600 k iterations while the bed only network only needs 450 k iterations. The medium short network has the largest number of instances, it takes more than 800 k iterations to converge.

### 4.5. Evaluation of the Whole System

The evaluation results are shown in Table 10. From the results, we can see that using the voxelized image based on the 3D instance segmentation output, the 3D object detection’s performance has a significant boosting, especially for the categories with a strong geometry shape such as toilet and bathtub. Overall, our Frustum Voxnet V2 has 84% of the detection performance compared to Frustum Pointnet. Our V2’s detection performance is improved by 11% compared to V1 based on RGBD images. Our depth only system can achieve 93% detection performance compared to RGBD-based system in Frustum Voxnet V2.

Table 11 shows the comparison of the number of parameters and inference time for our Frustum VoxNet V2 system and other systems. The instance segmentation time of our approach is faster than Frustum PointNets [9] as we have fewer parameters. When applying the pipeline technology, our system can have a pretty fast inference speed, making it applicable to systems requiring real-time inference.

### 4.6. Visualizations of 3D Segmentation Results

**Figure 18:**
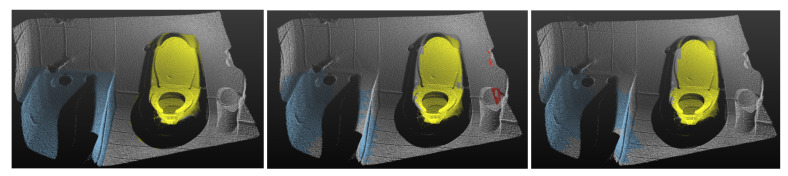
Visualizations of 3D segmentation results: Left is the ground truth. The middle is the prediction based on the frustum generated from the RGB color image. The image’s red color is the predicted segmentation for a false positive Chair during the 2D detection stage. Segmentation prediction based on the frustum generated from the DHS image is shown in the right image.

In Figure 18, we compare the 3D segmentation results of our system with the ground truth.

### 4.7. Visualizations of 3D Detection Results Compared between V2 and V1

**Figure 19:**
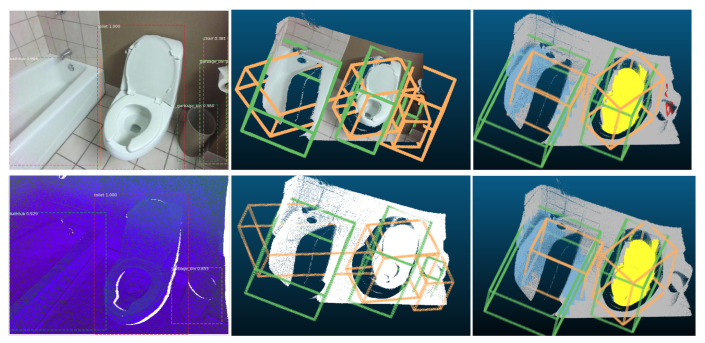
Visualizations of 3D detection results: In the upper one, the frustum is generated through the color image. In the bottom one, the frustum is generated based on DHS image. The middle is the 3D detection results from the Frustum VoxNet V1 and the right one is the results from Frustum VoxNet V2.

In Figure 19, we compare the 3D detection results between the Frustum VoxNet V1 and V2. From the results, we can find that using the instance segmentation results as the 3D detection network input can significantly improve the 3D detection result.

## 5. Conclusions

In this paper, we have provided novel, accurate, and efficient algorithms for solving the fundamental problems of 3D instance segmentation and object detection. We presented two 2D-based 3D detection systems by using 2D/3D CNNs. One of them named Frustum Voxnet V1 performs detection only and is faster, while the second named Frustum Voxnet V2 performs both segmentation and detection and it is more accurate. We integrate instance segmentation in our V2 system. The instance segmentation can give us a better understanding of the 3D image (depth or cloud point) and improve the final 3D object detection performance. Our V2’s detection performance is improved by 11% compared to V1 based on RGBD images. Our Frustum Voxnet V2 demonstrates comparable accuracy to the state-of-the-art (84% of the detection performance compared to Frustum PointNets [9]), but with more than two times improved run-time efficiency in 3D detection. This is due to the use of networks with fewer number of parameters than competing methods. It is also due to our ability to voxelize only parts of the 3D frustums. This leads to decreased memory requirements and improved resolution around the objects of interest. Our methods can operate in both depth only and RGB-D sensor modalities. Our depth only system can achieve 93% detection performance compared to RGBD-based system in Frustum Voxnet V2. We foresee that our methods will be used in real-time robotics applications. An avenue of future work could be integration of our system in a robotic platform.

Our main contributions are as follows:

- We have developed novel methods for 3D objection, classification, and instance segmentation. We have thoroughly tested their efficiency and accuracy as described in Section 3 and Section 4.
- We have significantly improved efficiency with respect to the state-of-the-art in 3D detection, as you can see in Table 4 and Table 10. Our 3D detection without segmentation has been presented in [14]. In this paper, we provide an enhanced system that performs both detection and segmentation. That improves the detection performance, as shown in Table 10, and it also includes instance segmentation results. The increased space and time efficiency makes our method appropriate for real-time robotic applications.
- We are able to provide accurate detection and segmentation results using depth only images, unlike competing methods such as [9], as you can see in Table 4 and Table 10. This is significant, since our methods can also work well in low lighting conditions, or with sensors that do not acquire RGB images.

## Figures and Tables

**Table 1:** Objects are classified into 4 categories based on there average physical size. Voxelization is processed based on each category.

	Short (h≤0.55)	Tall (h>0.55)
Small (max(w,h)≤0.3)	toilet	N/A
Medium (0.3<max(w,h)≤0.55)	chair, nightstand, sofa chair, garbage bin, bathtub	bookshef
Large (max(w,h)>0.55)	table, desk, sofa, bed, dresser	N/A

**Table 2:** Resolution and shape comparison between DeepSliding Shape [30] and ours. Anchors of the bed and trash can from [30] are used as examples of proposal’s physical size to make the comparison with ours.

Method	Network	3DCB Physical Size (m)	3DCB Shape	Resolution (cm)
DSS [30]	RPN	2.5×2.5×2.5	208×208×100	5.2×6.0×2.5
	Detection (bed)	6.7×6.7×3.2	30×30×30	2.0×2.0×0.95
	Detection (trash can)	1.0×1.0×1.2	30×30×30	0.3×0.3×0.5
Ours	small short	1.6×1.6×1.5	198×198×102	0.8×0.8×1.5
	medium short	3.2×3.2×1.7	198×198×102	1.6×1.6×1.7
	large short	4.8×4.8×2.2	198×198×102	2.4×2.4×2.2
	medium tall	2.8×2.8×3.0	134×134×134	2.1×2.1×2.2

**Table 3:** 2D detection results based on SUN RGB-D validation set. Evaluation metric is average precision with 2D Intersection over Union (IoU) threshold of 0.5.

	Bed	Toilet	Night Stand	Bathtub	Chair	Dresser	Sofa	Table	Desk	Bookshelf	Sofa Chair	Kitchen Counter	Kitchen Cabinet	Garbage Bin	Microwave	Sink
RGB-D RCNN [23] (RGB-D)	76.0	69.8	37.1	49.6	41.2	31.3	42.2	43.0	16.6	34.9	N/A	N/A	N/A	46.8	N/A	41.9
2D-driven [25] (RGB)	74.5	86.2	49.5	45.5	53.0	29.4	49.0	42.3	22.3	45.7	N/A	N/A	N/A	N/A	N/A	N/A
Frustum PointNets [9] (RGB)	56.7	43.5	37.2	81.3	64.1	33.3	57.4	49.9	77.8	67.2	N/A	N/A	N/A	N/A	N/A	N/A
OURS (RGB)	**81.0**	**89.5**	35.1	50.0	52.4	21.9	53.1	37.7	18.3	40.4	**47.8**	**22.0**	**29.8**	**52.8**	**39.7**	31.0
OURS (D)	**78.7**	77.6	34.2	51.9	51.8	16.5	48.5	34.9	14.2	19.2	48.7	19.1	18.5	30.3	22.2	30.1

**Table 4:** 3D detection results of system V1 on SUN RGB-D validation set. Evaluation metric is average precision with IoU threshold of 0.25 as proposed by [7]. Both Clouds of Oriented Gradients (COG)  [38] and 2D-driven [25] are using room layout context to boost performance while ours, Deep Sliding Shapes (DSS) [30], and Frustum PointNets [9] are not. Frustum PointNets [9] is using the 3D segmentation information to train the network to boost the 3D detection, while our system V1 and DSS [30] are not. Our system V2 uses the 3D segmentation information, and the results of V2 have a significant performance boost compared with V1.

	Bed	Toilet	Night Stand	Bathtub	Chair	Dresser	Sofa	Table	Desk	Bookshelf	Sofa Chair	Garbage Bin	Frustum Proposal Runtime	3D Detection Runtime	Total Runtime
DSS [30] (RGB-D)	78.8	78.9	15.4	44.2	61.2	6.4	53.5	50.3	20.5	11.9	N/A	N/A	N/A	N/A	19.55 s
COG [38] (RGB-D)	63.7	70.1	27.4	58.3	62.2	15.5	51.0	51.3	45.2	31.8	N/A	N/A	N/A	N/A	10–30 min
2D-driven [25] (RGB-D)	64.5	80.4	41.9	43.5	48.3	15.5	50.4	37.0	27.9	31.4	N/A	N/A	N/A	N/A	4.15 s
Frustum PointNets [9] (RGB-D)	81.1	90.0	58.1	43.3	64.2	32.0	61.1	51.1	24.7	33.3	N/A	N/A	**60 ms**	60 ms	**0.12 s**
OURS RGB-D (FPN+3D ResNetFCN6 V1)	78.5	84.5	34.5	42.4	47.2	18.2	40.3	30.4	12.4	18.0	47.1	47.6	110 ms	**48 ms**	0.16 s
OURS RGB-D (FPN+3D ResNetFCN35 V1)	79.5	84.6	36.2	44.6	49.1	19.6	40.8	27.5	12.5	19.1	47.9	**48.2**	110 ms	128 ms	0.24 s
OURS Depth only (FPN+3D ResNetFCN6 V1)	77.1	76.1	32.4	42.0	45.9	14.1	35.8	25.3	11.7	16.8	48.5	35.0	110 ms	**48 ms**	0.16 s
OURS Depth only (FPN+3D ResNetFCN35 V1)	77.4	76.8	33.1	43.7	45.8	15.2	37.3	25.5	11.8	17.4	**48.8**	35.4	110 ms	148 ms	0.24 s

**Table 5:** 2D/3D detection results based on YOLO v3 vs. FPN. 2D detection is based on RGB images. 3D detection is based on RGB-D images and the 3D detection network is based on Frustum VoxNet V1.

	2D Network	3D Network	Bed	Toilet	Chair	Sofa	Table
2D Detection	FPN		81.0	89.5	52.4	53.1	37.7
	YOLO v3		71.8	73.7	38.5	51.4	22.1
3D Detection	FPN	3D ResNetFCN6	78.5	84.5	47.2	40.3	30.4
	YOLO v3	3D ResNetFCN6	66.9	69.8	30.1	37.9	18.8

**Table 6:** Number of parameters and inference time comparison between Frustum Pointnet and our system. For YOLO v3, input resolution is 416 by 416 and the model FLOPS is 65.86 Bn.

Methods	# Parameters		Runtime (ms)		
—–	**Frustum** **Proposal**	**3D** **Detection**	**Frustum** **Proposal**	**3D** **Detection**	**Total**
Frustum PointNets (FPN + Pointnet V1)	**28 M**	19 M	60	60	120
Frustum PointNets (FPN + Pointnet V2)	**28 M**	22 M	60	107	167
Ours w/o Pipeline (FPN + 3D ResNetFCN6 V1)	42 M	**2.5 M**	110	**48**	158
Ours w/o Pipeline (FPN + 3D ResNetFCN35 V1)	42 M	23.5 M	110	149	259
Ours w/o Pipeline (YOLO v3 + 3D ResNetFCN6 V1)	N/A	**2.5 M**	**29**	**48**	**77**
Ours with Pipeline (YOLO v3 + 3D ResNetFCN6 V1)	N/A	**2.5 M**	**29**	**48**	**48**

**Table 7:** Result comparison between average and predicted center from Frustum VoxNet.

		$\bar{x-x^{*}}$	$\bar{y-y^{*}}$	$\bar{z-z^{*}}$	$\bar{D_{\mathrm{xyz}}}$
Table	Frustum Average Center	−0.005	−0.233	0.075	0.522
	Predicted from Frustum VoxNet	0.014	−0.040	0.030	**0.395**
Desk	Frustum Average Center	−0.010	−0.198	0.109	0.428
	Predicted from Frustum VoxNet	0.028	−0.040	0.048	**0.319**
Sofa	Frustum Average Center	−0.015	−0.168	0.010	0.516
	Predicted from Frustum VoxNet	0.007	0.041	0.013	**0.444**
Bed	Frustum Average Center	0.031	−0.195	0.013	0.573
	Predicted from Frustum VoxNet	−0.009	0.010	−0.012	**0.354**

**Table 8:** Detailed evaluation results. Frustum VoxNet is evaluated based on SUN RGB-D validation set. Frustums used to finalize detection are generated from ground truth 2D bounding boxes. The 3D IoU threshold used for 3D recall is 0.25.

Category	Instance Number	$\bar{D_{x}}$	$\bar{D_{y}}$	$\bar{D_{z}}$	$\bar{D_{\mathrm{xyz}}}$	$\bar{D_{w}}$	$\bar{D_{d}}$	$\bar{D_{h}}$	$\bar{D_{\mathrm{wdh}}}$	$\bar{|o^{*}\cdot o|}$	Average 3D IoU	3D Recall (IoU@0.25)
table	1269	0.201	0.280	0.070	0.395	0.206	0.132	0.042	0.287	0.747	0.319	0.656
desk	457	0.158	0.220	0.080	0.319	0.180	0.122	0.052	0.258	0.752	0.329	0.674
dresser	91	0.248	0.298	0.135	0.489	0.126	0.064	0.107	0.209	0.758	0.241	0.451
sofa	381	0.213	0.320	0.075	0.444	0.210	0.099	0.048	0.264	0.847	0.459	0.796
bed	441	0.195	0.220	0.096	0.354	0.154	0.125	0.083	0.246	0.746	0.462	0.898
night stand	220	0.156	0.226	0.069	0.314	0.050	0.037	0.044	0.087	0.830	0.329	0.655
bathtub	37	0.162	0.114	0.067	0.226	0.134	0.071	0.040	0.173	0.805	0.383	0.811
chair	4777	0.118	0.217	0.067	0.286	0.038	0.048	0.047	0.089	0.886	0.369	0.708
sofa chair	575	0.109	0.168	0.070	0.242	0.058	0.051	0.045	0.103	0.840	0.466	0.849
garbage bin	248	0.065	0.098	0.050	0.145	0.043	0.035	0.042	0.082	0.760	0.384	0.782
toilet	87	0.051	0.093	0.073	0.148	0.028	0.039	0.047	0.076	0.825	0.498	0.929
bookshelf	106	0.183	0.303	0.130	0.433	0.410	0.063	0.149	0.474	0.880	0.345	0.679

**Table 9:** Input and output shapes for the segmentation network based on different scale networks. We combine the table and desk into the same category, so we have the number of categories for the medium short is 4 instead of 5.

Network	3DCB Physical Size (m)	Input 3DCB Shape	Output Tensor Shape
small short	1.6×1.6×1.5	198×198×102	48×48×24×1
medium short	3.2×3.2×1.7	198×198×102	48×48×24×4
large short	4.8×4.8×2.2	198×198×102	48×48×24×4
medium tall	2.8×2.8×3.0	134×134×134	32×32×32×1

**Table 10:** 3D detection results on SUN RGB-D validation set. Evaluation metric is average precision with IoU threshold of 0.25 as proposed by [7]. Average mAP is used to have an overall comparison.

	Bed	Toilet	Night Stand	Bathtub	Chair	Dresser	Sofa	Table	Desk	Bookshelf	Average mAP	Frustum Proposal Runtime	3D Detection Runtime	Total Runtime
Frustum PointNets [9] (RGB-D)	81.1	90.0	58.1	43.3	64.2	32.0	61.1	51.1	24.7	33.3	53.4	**60 ms**	60 ms	**0.12 s**
OURS RGB-D (FPN+3D ResNetFCN6 V1)	78.5	84.5	34.5	42.4	47.2	18.2	40.3	30.4	12.4	18.0	40.6	110 ms	**48 ms**	0.16 s
OURS RGB-D (FPN+3D ResNetFCN6 V2)	79.9	**91.6**	38.8	56.7	48.1	22.3	43.2	34.1	15.1	19.8	45.0	110 ms	100 ms	0.21 s
OURS Depth only (FPN+3D ResNetFCN6 V1)	77.1	76.1	32.4	42.0	45.9	14.1	35.8	25.3	11.7	16.8	37.7	110 ms	**48 ms**	0.16 s
OURS Depth only (FPN+3D ResNetFCN6 V2)	78.6	89.0	37.2	45.7	46.3	20.3	37.0	32.5	12.9	17.7	41.7	110ms	100 ms	0.21 s

**Table 11:** Number of parameters and inference time comparison between Frustum Pointnet and our systems V1 and V2. For YOLO v3, input resolution is 416 by 416 and the model FLOPS is 65.86 Bn.

Methods	# Parameters		Runtime (ms)			
—–	**Frustum** **Proposal**	**3D** **Detection**	**Frustum** **Proposal**	**3D Instance** **Segmentation**	**3D** **Detection**	**Total**
Frustum PointNets [9] (FPN + Pointnet V1)	**28 M**	19 M	60	-	60	120
Frustum PointNets [9] (FPN + Pointnet V2)	**28 M**	22 M	60	88	19	167
Ours w/o Pipeline (FPN + 3D ResNetFCN6 V1)	42 M	**2.5 M**	110	-	**48**	158
Ours w/o Pipeline (FPN + 3D ResNetFCN6 V2)	42 M	5.5 M	110	52	48	210
Ours w/o Pipeline (YOLO v3 + 3D ResNetFCN6 V1)	N/A	**2.5 M**	**29**	-	48	**77**
Ours with Pipeline (YOLO v3 + 3D ResNetFCN6 V1)	N/A	**2.5 M**	**29**	-	48	**48**
Ours w/o Pipeline (YOLO v3 + 3D ResNetFCN6 V2)	N/A	5.5 M	**29**	**52**	48	129
Ours with Pipeline (YOLO v3 + 3D ResNetFCN6 V2)	N/A	5.5 M	**29**	**52**	48	**52**

## Data Availability

The data presented is available from rgbd.cs.princeton.edu.

## Abbreviations

The following abbreviations are used in this manuscript:

3DBBOX	3D Bounding Box
3DCB	3D Cropped Box
BEV	Bird’s Eye View
BN	Batch Normalization
CNN	Convolutional Neural Network
DHS	Depth Height and Signed angle
FCN	Fully Convolutional Neural Network
FPN	Feature Pyramid Network
GN	Group Normalization
IoI	Intersection over Itself
IoU	Intersection over Union
SGD	Stochastic Gradient Descent
YOLO	You Only Look Once
